# RBM15-mediated m^6^A modification in cancer progression and tumor immunity: molecular mechanisms and therapeutic potential

**DOI:** 10.3389/fcell.2025.1713615

**Published:** 2025-12-01

**Authors:** Chunhong Li, Xiulin Jiang, Yixiao Yuan, Qiang Wang

**Affiliations:** 1 Department of Oncology, Suining Central Hospital, Suining, China; 2 Department of Systems Biology, City of Hope Comprehensive Cancer Center Biomedical Research Center, Monrovia, CA, United States; 3 Department of Gastrointestinal Surgical Unit, Suining Central Hospital, Suining, China

**Keywords:** RBM15, RNA-binding protein, m6A modification, epitranscriptome, cancer progression, tumor immunity, biomarker, therapeutic target

## Abstract

RBM15, a key RNA-binding protein involved in m^6^A RNA methylation, plays multifaceted roles in cancer development and tumor immunity. Emerging evidence indicates that RBM15 regulates tumor cell proliferation, migration, invasion, and metabolic reprogramming through modulation of RNA stability, splicing, and translation. Beyond its tumor-intrinsic effects, RBM15 influences the tumor immune microenvironment by affecting immune cell differentiation, activation, and cytokine production, thereby contributing to immune evasion. Dysregulated RBM15 expression has been observed across various cancer types, correlating with poor prognosis and therapy resistance. These findings highlight RBM15 as a promising biomarker for cancer diagnosis and prognosis and suggest its potential as a therapeutic target. Future studies focusing on RBM15-targeted interventions, either alone or in combination with immunotherapy, may provide novel strategies for precision cancer treatment.

## 1 Introduction

RNA-binding proteins (RBPs) are a class of regulatory factors that specifically recognize and bind RNA molecules, participating extensively in multiple layers of post-transcriptional regulation, including RNA splicing, modification, stability, localization, and translation (ME, 2022). Accumulating evidence has demonstrated that RBPs play central roles in the dynamic regulation of gene expression and are involved in diverse physiological and pathological processes by influencing cell fate determination, metabolic reprogramming, and signal transduction (Brownmiller and Caplen, 2023). During tumorigenesis and cancer progression, aberrant expression or dysfunction of RBPs frequently leads to an imbalance in oncogene and tumor suppressor gene expression, thereby promoting uncontrolled cell proliferation, inhibiting apoptosis, enhancing invasion and metastasis, and shaping an immune-evasive tumor microenvironment (Wang et al., 2022; Li et al., 2022). Consequently, RBPs have increasingly become a major focus in both cancer biology and clinical research.

RNA-binding motif protein 15 (RBM15), an important member of the RBP family, belongs to the SPEN (Split ends family) group and shares a certain degree of structural and functional overlap with its homolog RBM15B (Hu et al., 2016; Jiang et al., 2021). RBM15 contains canonical RNA recognition motifs (RRMs) as well as transcriptional regulatory domains, enabling it to interact with various classes of RNA molecules and transcriptional complexes (Hu et al., 2016). RBM15 and its paralog RBM15B are RNA-binding proteins that recruit the m^6^A methyltransferase complex to specific target RNAs, thereby facilitating m^6^A modification (Patil et al., 2016). Through this mechanism, RBM15 indirectly regulates m^6^A-mediated processes, including mRNA splicing, stability, and translation. While RBM15 does not directly engage with the translation machinery, m^6^A-marked transcripts can be recognized by reader proteins such as YTHDF1, which promote translation, highlighting an indirect role for RBM15 in modulating protein synthesis (Wang et al., 2024). Through these interactions, RBM15 participates not only in post-transcriptional regulation but also in chromatin-level gene expression control. Importantly, RBM15 plays a pivotal role in hematopoietic system development, and its dysregulation has been strongly linked to hematological malignancies such as acute myeloid leukemia (AML) (Raffel et al., 2007; Niu et al., 2009). With the emergence of epitranscriptomics, the unique role of RBM15 in RNA modification has been further elucidated. As a critical regulator of N6-methyladenosine (m^6^A) modification, RBM15 recruits the methyltransferase complex (comprising core components such as METTL3, METTL14, and WTAP) to specific RNA regions, thereby determining the deposition pattern of m^6^A (Patil et al., 2016; Dou et al., 2023a). Through this mechanism, RBM15 exerts profound effects on the stability, alternative splicing, and translational efficiency of target mRNAs and non-coding RNAs, all of which are intimately associated with tumorigenesis and cancer progression.

In fact, RBM15 has been reported to participate in multiple key biological processes during cancer development (Cheng et al., 2024). For instance, in hematological malignancies, the RBM15-MKL1 fusion protein generated by chromosomal translocation can drive leukemogenesis (Mayday et al., 2025). In various solid tumors, including hepatocellular carcinoma, breast cancer, gastric cancer, and lung cancer, aberrant RBM15 expression has been correlated with enhanced proliferation, resistance to apoptosis, increased metastatic potential, and poor patient prognosis (Cao et al., 2024). Moreover, RBM15 indirectly modulates the balance between oncogenes and tumor suppressor genes by orchestrating m^6^A modification networks of specific mRNAs and non-coding RNAs (Zhang J. et al., 2023).

Of particular note, the role of RBM15 in tumor immunity has recently attracted growing attention. Studies have shown that RBM15, through m^6^A modification and post-transcriptional regulation, can influence the expression of immune-related genes, thereby modulating antigen processing and presentation, cytokine secretion, and the expression of immune checkpoint molecules (Wang et al., 2025a). These changes, in turn, affect the infiltration and functional states of immune cells within the tumor microenvironment. This indicates that RBM15 not only drives intrinsic malignant behavior of tumor cells but may also promote immune evasion by shaping an immunosuppressive microenvironment, ultimately influencing the efficacy of immunotherapy.

Taken together, RBM15 serves as a crucial link between epitranscriptomic regulation and cancer biology, exerting multilayered functions through diverse mechanisms. Previous reviews on RBM15 have primarily focused on its roles in cancer biology, with limited discussion of its functions in the immune system. In contrast, this review aims to systematically summarize recent advances regarding the roles of RBM15 in cancer progression and tumor immunity, with a particular emphasis on its m^6^A-mediated molecular mechanisms and potential clinical applications. We hope to provide new perspectives for understanding the mechanistic basis of RBM15 in tumorigenesis and progression, and to establish a theoretical foundation for its development as both a prognostic biomarker and a therapeutic target.

## 2 Molecular structure and biological functions of RBM15

RBM15 is an important member of the RNA-binding protein family and belongs to the SPEN family. It shares high structural and functional similarity with its homolog RBM15B (Hu et al., 2016). The RBM15 protein is composed of multiple domains, including canonical RRMs and a C-terminal SPEN paralog and ortholog C-terminal (SPOC) domain. The RRM domains enable RBM15 to specifically bind diverse RNA molecules, including both mRNAs and non-coding RNAs, thereby participating in RNA processing and modification (Ma et al., 2024). Meanwhile, the SPOC domain interacts with transcription factors and epigenetic regulators, thereby mediating transcriptional repression, chromatin remodeling, and signal regulation. This multi-domain architecture endows RBM15 with dual properties of RNA binding and transcriptional regulation, placing it in a central position within the gene expression regulatory network (Patil et al., 2016).

At the post-transcriptional level, RBM15 participates in a variety of RNA processing and metabolic processes. It can bind precursor mRNAs and regulate alternative splicing, thereby influencing the production and function of different isoforms (Wang et al., 2024). By facilitating mRNA nuclear export, RBM15 ensures that transcripts of certain key genes are efficiently transported to the cytoplasm for translation. In addition, RBM15 regulates transcript stability and half-life, thereby modulating gene expression strength (Shi et al., 2024). Furthermore, RBM15 interacts with translation factors and translation-regulatory complexes to influence protein synthesis. Collectively, these functions establish RBM15 as a crucial regulator of cell proliferation, differentiation, and stress responses.

In addition to RBM15, several other m^6^A methyltransferases, including METTL3, METTL14, and WTAP, play critical roles in tumor immunity by modulating RNA methylation and downstream immune responses (Song et al., 2023). While METTL3 and METTL14 broadly regulate m^6^A deposition and influence immune cell differentiation and cytokine expression, RBM15 functions more selectively by recruiting the m^6^A machinery to specific transcripts, thereby affecting the splicing, stability, and translation of immune-relevant genes (Dou et al., 2023b). Emerging evidence suggests that, compared with canonical methyltransferases, RBM15 may exert a more targeted influence on immune cell fate and anti-tumor responses, highlighting its unique and potentially complementary role in shaping tumor immunity (Wang et al., 2025a).

Among its diverse roles, the most prominent function of RBM15 lies in its interaction with the m^6^A methyltransferase complex. Studies have shown that RBM15 can recognize specific RNA sequences via its RRM domains and recruit the core m^6^A methyltransferase complex, consisting of METTL3, METTL14, and WTAP, to target RNA sites for methylation (Patil et al., 2016). RBM15 not only provides targeting specificity but also enhances complex assembly and enzymatic activity through interaction with WTAP, thereby improving the efficiency of m^6^A modification. The resultant changes in RNA modification markedly influence mRNA stability, splicing patterns, and translational efficiency, which are further mediated by m^6^A reader proteins such as the YTH family (Zhang J. et al., 2023). Thus, by precisely controlling the distribution and levels of m^6^A modification, RBM15 plays a pivotal role in epitranscriptomic regulation and profoundly affects the expression and function of cancer-associated genes.

## 3 RBM15-mediated epitranscriptomic modifications and tumor regulatory mechanisms

RNA modifications have emerged as an essential component of epitranscriptomics and have received increasing attention in cancer research (Roundtree et al., 2017). Among them, m^6^A is the most extensively studied RNA modification, widely distributed across mRNAs and non-coding RNAs, and regulating their stability, splicing, nuclear export, and translation efficiency (An and Duan, 2022). As a critical regulator of m^6^A modification, RBM15 influences tumor-associated gene expression not only through m^6^A-dependent mechanisms but also via m^6^A-independent pathways of post-transcriptional regulation.

### 3.1 m^6^A-dependent mechanisms

RBM15 interacts with WTAP, METTL3, and METTL14 within the m^6^A methyltransferase complex, recruiting the complex to specific RNA regions and determining the m^6^A modification landscape of target RNAs (Patil et al., 2016). This process exerts decisive effects on the expression of numerous cancer-related genes. Regulation of mRNA stability: RBM15 promotes m^6^A modification on oncogenic mRNAs, thereby enhancing their stability and half-life, leading to sustained oncogene expression and promoting cell proliferation and survival (Wang et al., 2022). Conversely, for certain tumor suppressor genes, RBM15-mediated m^6^A modification can accelerate their degradation, facilitating tumorigenesis. Regulation of translation efficiency: m^6^A functions as a “translational switch” that, through recognition by m^6^A reader proteins such as YTHDF1/3, promotes efficient translation of target mRNAs. By modulating this process, RBM15 indirectly enhances the activity of signaling pathways that drive tumor growth and metastasis.

### 3.2 m^6^A-independent mechanisms

In addition to m^6^A modification, RBM15 contributes to tumor regulation through multiple m6^6^-independent pathways:Alternative splicing regulation: By directly binding to specific precursor mRNAs through its RRMs, RBM15 regulates alternative splicing events, altering isoform ratios and thereby modulating oncogenic signaling pathways (Hu et al., 2016). Nuclear export control: RBM15 facilitates nuclear export of specific cancer-associated mRNAs, ensuring proper subcellular localization and subsequent protein expression. Polyadenylation site selection: RBM15 is also involved in regulating 3′end polyadenylation site usage, which influences transcript stability and translational potential (Horiuchi et al., 2021). This mechanism has been shown to play roles in hematopoiesis and cancer development.

### 3.3 Cooperation with epigenetic and transcription factors

RBM15 functions not in isolation but within cooperative regulatory networks involving epigenetic factors and transcription factors, amplifying its role in cancer progression. For example, RBM15 interacts with WDR5 to couple chromatin modifications with transcriptional activation, thereby influencing oncogene regulation (Dou et al., 2023a; Schwaemmle et al., 2025). RBM15-mediated m^6^A modifications require recognition by YTH family proteins, which execute distinct functional outcomes: YTHDF1 enhances translation, whereas YTHDF2 promotes mRNA degradation, both relying on RBM15-mediated site-specific recruitment (Wang et al., 2024). Moreover, RBM15 collaborates with SPEN family proteins and other transcriptional repressors to form integrated networks spanning transcriptional and post-transcriptional regulation, reinforcing its multilayered impact on tumorigenesis (Moindrot et al., 2015). Collectively, RBM15 integrates m^6^A-dependent and -independent mechanisms with epitranscriptomic and epigenetic networks, establishing itself as a central hub in tumor progression.

### 3.4 Upstream regulatory mechanisms of RBM15

**FIGURE 1:**
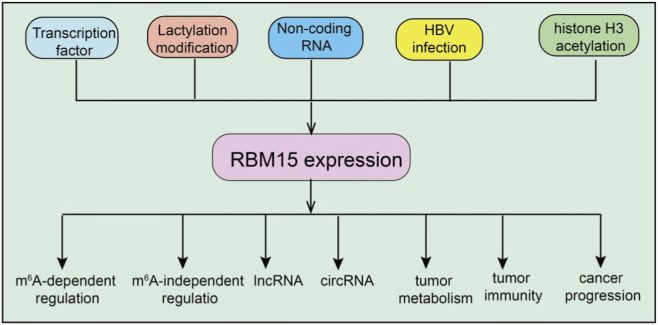
Regulation of RBM15 expression and its downstream effects. Multiple upstream factors, including transcription factors, lactylation modification, non-coding RNAs, HBV infection, and histone H3 acetylation, regulate RBM15 expression. RBM15 in turn influences diverse cellular processes through both m^6^A-dependent and m6A-independent regulation, affecting lncRNAs, circRNAs, tumor metabolism, tumor immunity, and cancer progression.

The expression and function of RBM15 are finely regulated by diverse upstream signals and epigenetic modifications, and these regulatory mechanisms display considerable variability across different cancer types (Figure 1). For instance, in lung adenocarcinoma, tumor cells uptake lactate through monocarboxylate transporter 1 (MCT1), which promotes lactylation of RBM15 at the K850 residue (Zhao et al., 2025). This lactylation not only prevents RBM15 protein degradation but also enhances its interaction with the m^6^A methyltransferase METTL3, thereby elevating global m^6^A levels and further driving tumor cell proliferation and migration. Notably, mutation at the K850 site markedly attenuates the oncogenic potential of RBM15, indicating that lactylation serves as a critical regulatory mechanism for its stability and activity (Zhao et al., 2025). In virus-associated cancers, RBM15 regulation is closely linked to viral proteins. Hepatitis B virus (HBV) infection upregulates RBM15 expression through its X protein (HBx), thereby contributing to the pathogenesis of hepatocellular carcinoma (Xu et al., 2025). Similarly, in cervical cancer, the HPV E6 protein inhibits autophagy-dependent degradation of RBM15, leading to intracellular protein accumulation without affecting its mRNA levels (Nie et al., 2023). These findings suggest that viral proteins can indirectly enhance RBM15 function by modulating protein homeostasis. In addition, in clear cell renal cell carcinoma, high RBM15 expression is mainly attributed to histone H3 acetylation at its promoter region, mediated by the histone acetyltransferases EP300/CBP (Zeng et al., 2022). RBM15 is methylated at R578 by PRMT1, a modification that triggers its ubiquitin-mediated degradation via the E3 ligase CNOT4. This post-translational modification reduces RBM15 protein levels, impairing megakaryocyte differentiation by altering alternative splicing of key genes such as GATA1, RUNX1, TAL1, and c-MPL (Zhang et al., 2015). Restoring RBM15 levels rescues differentiation, highlighting the critical regulatory role of this methylation (Zhang et al., 2015). Taken together, these studies demonstrate that the upstream regulation of RBM15 involves multilayered mechanisms, including metabolite-mediated post-translational modifications, viral protein–induced modulation of protein stability, and epigenetic activation of transcription. Such precise regulation of RBM15 expression and activity across distinct cancer types ultimately drives tumor initiation and progression.

## 4 Functions and mechanisms of RBM15 in cancer progression

**FIGURE 2:**
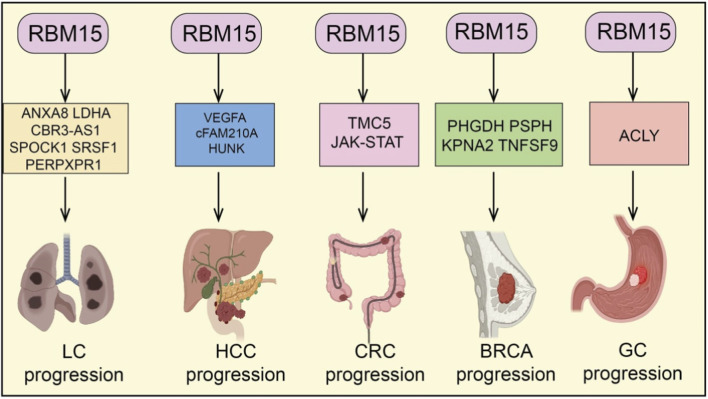
RBM15 in multiple solid tumors. RBM15 regulates different downstream genes and pathways to promote cancer progression: LC (lung cancer) via ANXA8, LDHA, CBR3-AS1, SPOCK1, SRSF1, and PERPXPR1; HCC (hepatocellular carcinoma) via VEGFA, cFAM210A, and HUNK; CRC (colorectal cancer) via TMC5 and JAK-STAT signaling; BRCA (breast cancer) via PHGDH, PSPH, KPNA2, and TNFSF9; and GC (gastric cancer) via ACLY.

**FIGURE 3:**
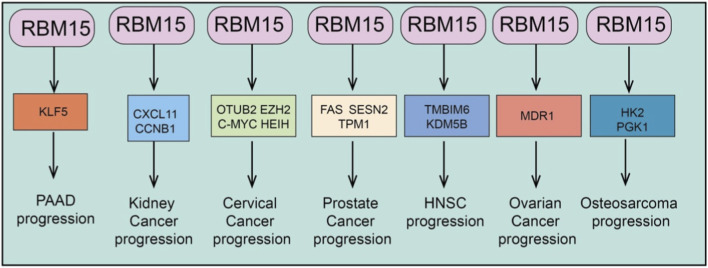
Broad oncogenic functions of RBM15 across cancer types. RBM15 promotes tumor progression by regulating distinct targets across multiple cancers: PAAD (pancreatic adenocarcinoma) via KLF5; Kidney cancer via CXCL11 and CCNB1; Cervical cancer via OTUB2, EZH2, c-MYC, and HEIH; Prostate cancer via FAS, SESN2, and TPM1; HNSC (head and neck squamous carcinoma) via TMBIM6 and KDM5B; Ovarian cancer via MDR1; and Osteosarcoma via HK2 and PGK1.

RBM15, as an important m^6^A RNA methyltransferase, plays a critical role in tumorigenesis and progression across various cancer types (Hu et al., 2016). Numerous studies have demonstrated that RBM15 is broadly overexpressed in tumors of different tissue origins. Its aberrant upregulation not only promotes cancer cell proliferation, migration, invasion, and drug resistance but also drives malignant phenotypes through regulating multiple biological processes, including cell cycle progression, apoptosis, ferroptosis, cuproptosis, epithelial–mesenchymal transition (EMT), immune cell infiltration, and metabolic pathways (Mayday et al., 2025). Mechanistically, RBM15 enhances the stability and expression of key mRNAs or non-coding RNAs via m^6^A modification, thereby mediating diverse downstream signaling axes (Wang et al., 2025a). This establishes multilayered oncogenic networks that further promote tumor growth and therapeutic resistance. Therefore, RBM15 functions not only as a pivotal molecular regulator in cancer development but also as a promising therapeutic target for potential clinical intervention (Figures 2, 3).

### 4.1 Lung cancer

In lung cancer, accumulating evidence has demonstrated that RBM15 acts as a pro-tumorigenic factor that drives malignant progression, particularly in non-small cell lung cancer (NSCLC). Functionally, RBM15 enhances cell proliferation, invasion, and migration. Mechanistically, RBM15 regulates YTHDF1/YTHDF2-mediated signaling, upregulates KLF1, and suppresses TRIM13, thereby promoting the expression of ANXA8 (Wang et al., 2024). Elevated KLF1 or reduced TRIM13 can partially offset the inhibitory effects caused by RBM15 depletion, while ANXA8 overexpression diminishes the tumor-suppressive impact of RBM15 silencing on malignant behaviors. *In vivo* studies further confirmed that RBM15 promotes NSCLC cell proliferation and metastasis via the KLF1–TRIM13/ANXA8 signaling axis (Wang et al., 2024). Collectively, these findings highlight RBM15 as a pivotal driver of NSCLC aggressiveness through a KLF1-centered regulatory pathway. RBM15 is consistently overexpressed in lung cancer cells, and its silencing markedly reduces cell viability, suppresses proliferation, invasion, and migration, while also restraining tumor growth in xenograft mouse models. Mechanistic investigations revealed that RBM15 depletion modulates ferroptosis-related genes, leading to an increase in intracellular free iron (Fe^2+^), lipid peroxidation, and lipid hydroperoxides. Furthermore, RBM15 knockdown diminishes the activity of the TGF-β/Smad2 pathway, and pharmacological activation of TGF-β signaling with SRI-011381 reverses the inhibitory effect of RBM15 silencing on tumor cell growth (Feng et al., 2023). These findings indicate that RBM15 promotes lung cancer progression through dual regulation of ferroptosis and the TGF-β/Smad2 signaling pathway. In lung adenocarcinoma (LUAD), RBM15 is markedly upregulated and promotes proliferation by stabilizing LDHA mRNA through m^6^A modification. Elevated RBM15 expression correlates with poor prognosis, suggesting the RBM15/LDHA axis as a potential therapeutic target (Shi et al., 2024). Moreover, in NSCLC, RBM15 facilitates radioresistance through an m^6^A-IGF2BP3-dependent mechanism that upregulates CBR3-AS1, which in turn regulates the miR-409–3p/CXCL1 axis, promoting myeloid-derived suppressor cell (MDSC) recruitment and suppressing T cell activity (Hu et al., 2025). Silencing RBM15 reverses this process, thereby enhancing radiosensitivity and improving immune infiltration. Together, these results underscore RBM15 as a mediator of both metabolic reprogramming and immune evasion in lung cancer. Metabolic regulation further modulates RBM15 activity. In LUAD, L-lactate imported via MCT1 promotes RBM15 lactylation at lysine 850, which inhibits its degradation and strengthens its interaction with METTL3 (Zhao et al., 2025). This modification increases global m^6^A levels, thereby enhancing cancer cell proliferation and migration. Mutation at the K850 site significantly weakens RBM15’s oncogenic function, demonstrating that lactylation is a critical regulatory mechanism for RBM15 stability and activity (Zhao et al., 2025). RBM15 expression is particularly elevated in LUAD harboring EGFR mutations, where it promotes proliferation and migration and contributes to osimertinib resistance by enhancing SPOCK1 mRNA m^6^A modification and driving EMT. The RBM15–SPOCK1 axis is activated in resistant cells, suggesting that early therapeutic targeting of RBM15 may improve EGFR-TKI treatment efficacy (Li et al., 2025). In NSCLC, RBM15 also stabilizes SRSF1 through m^6^A modification and recruits YTHDF3, promoting exon 21 alternative splicing of ATP7B. This inhibits cuproptosis and enhances tumor cell proliferation and invasion, while RBM15 inhibition triggers cuproptosis and suppresses tumor growth. Additional studies have identified novel downstream targets of RBM15 (Mao et al., 2025). In LUAD, RBM15 promotes PERP mRNA stability via m^6^A modification, thereby suppressing the p53 signaling pathway and enhancing cell proliferation, migration, and invasion. PERP silencing reverses the malignant phenotypes driven by RBM15, highlighting the RBM15/PERP axis as a critical regulator of LUAD progression (Li et al., 2024). Similarly, RBM15 stabilizes XPR1 mRNA through m^6^A modification, which promotes proliferation and invasion while suppressing apoptosis, oxidative stress, and ferroptosis (Sun et al., 2025). RBM15 knockdown impedes tumor growth via XPR1 regulation, underscoring the oncogenic role of the RBM15/XPR1 axis in LUAD. In summary, RBM15 is markedly overexpressed in NSCLC and LUAD, where it regulates diverse biological processes—including ferroptosis, cuproptosis, metabolic reprogramming, EMT, and immune modulation—through distinct m^6^A-dependent signaling pathways. These findings collectively position RBM15 as a central oncogenic hub and a promising therapeutic target in lung cancer.

### 4.2 Liver cancer

In hepatocellular carcinoma (HCC), RBM15 plays a vital role in tumor progression, particularly in regulating angiogenesis. Vascular endothelial growth factor A (VEGFA) is highly expressed in HCC and promotes tumor vascularization. Studies have shown that VEGFA undergoes extensive m^6^A hypermethylation mediated by RBM15, a component of the methyltransferase complex. The m^6^A-marked VEGFA mRNA is recognized and stabilized by IGF2BP3 and YTHDF2, thereby enhancing VEGFA expression and its downstream functions, such as human umbilical vein endothelial cell (HUVEC) migration and tube formation (Xu et al., 2024). In xenograft models, silencing RBM15, IGF2BP3, or YTHDF2 significantly reduces VEGFA expression and suppresses tumor growth. Clinical analyses further reveal a positive correlation between VEGFA expression and RBM15/IGF2BP3/YTHDF2 levels, underscoring the central role of RBM15-mediated m^6^A modification in VEGFA regulation and tumor angiogenesis (Xu et al., 2024). Thus, RBM15 may serve as a potential target for anti-angiogenic therapy in HCC. In HBV-related HCC, viral proteins modulate RBM15-mediated RNA regulation. HBx upregulates RBM15 and enhances the m^6^A modification of circular RNA cFAM210A, leading to its degradation through the YTHDF2–HRSP12–RNase P/MRP pathway (Yu et al., 2023). Notably, cFAM210A is downregulated in HCC and negatively correlates with tumorigenesis, as it suppresses cell proliferation, stemness maintenance, and tumor formation. Mechanistically, cFAM210A binds YBX1 and inhibits its phosphorylation, thereby suppressing YBX1-mediated transcriptional activation of MET. These findings suggest that HBx promotes hepatocarcinogenesis by inducing RBM15-dependent cFAM210A degradation, while cFAM210A itself represents a potential therapeutic target for HBV-related HCC (Yu et al., 2023). In summary, RBM15 drives HCC progression by enhancing angiogenesis through VEGFA stabilization and by mediating HBx-induced circRNA degradation, collectively establishing RBM15 as both a tumor promoter and a potential therapeutic target in liver cancer.

### 4.3 Colon cancer

In colon adenocarcinoma (COAD), RBM15 expression is markedly elevated and contributes to tumor progression. ITGBL1, a downstream effector, is also highly expressed. Functional studies demonstrate that ITGBL1 knockdown inhibits COAD cell proliferation, migration, and invasion, while reducing M2 macrophage polarization and enhancing lymphocyte-mediated immunity (Zhu et al., 2025). Importantly, RBM15 deletion suppresses tumor growth *in vivo*. Mechanistic studies reveal that RBM15 upregulates ITGBL1 expression through m^6^A methylation, thereby promoting malignant progression and immune suppression in COAD (Zhu et al., 2025). In addition, TMC5 expression is significantly upregulated in both COAD tissues and cells, in parallel with RBM15 (Tian et al., 2025). Silencing TMC5 inhibits COAD proliferation, migration, invasion, EMT, and glycolysis, while simultaneously inducing apoptosis and ferroptosis, and suppressing tumor growth *in vivo*. Mechanistic studies show that RBM15 maintains TMC5 RNA stability and expression via m^6^A modification, thereby promoting COAD malignancy (Tian et al., 2025). Broader genomic analyses of colon cancer (CC) have identified RBM15 among five critical m^6^A-related genes, including WTAP, CBLL1, and YTHDC2. RBM15 is consistently upregulated, and its silencing markedly suppresses CC cell proliferation, migration, invasion, and tumor formation. Moreover, RBM15 depletion downregulates JAK–STAT pathway proteins, suggesting that RBM15 promotes colon cancer progression via JAK–STAT signaling (Zhang C. et al., 2023). In summary, RBM15 promotes colon cancer development through m^6^A-dependent stabilization of oncogenic targets such as ITGBL1 and TMC5, while activating the JAK–STAT signaling pathway. These findings highlight RBM15 as both a prognostic biomarker and a promising therapeutic target in COAD.

### 4.4 Breast cancer

In breast cancer, RBM15 exerts profound oncogenic functions, particularly in basal-like and triple-negative subtypes. In basal-like breast cancer, RBM15 is markedly upregulated and associated with poor clinical prognosis (Liu et al., 2024). Mechanistic studies reveal that RBM15 directly binds to RNA transcripts and regulates the m^6^A modification of serine and glycine metabolism–related genes, including PHGDH, PSAT1, PSPH, and SHMT2 (Park et al., 2024). This regulation promotes tumor cell growth by reprogramming amino acid metabolism. Notably, the tumor-promoting effects of RBM15 largely depend on enhanced serine–glycine metabolism, underscoring its role as a metabolic regulator and therapeutic target in breast cancer. In breast cancer tissues and cells, both RBM15 and KPNA2 are highly expressed. Functional experiments demonstrate that RBM15 silencing suppresses proliferation, migration, invasion, and immune modulation, while inhibiting tumor growth *in vivo* (Wang et al., 2025a). Mechanistically, RBM15 maintains KPNA2 mRNA stability via m^6^A modification, thereby driving malignant progression. RBM15 also contributes to chemoresistance (Wang et al., 2025a). In paclitaxel (PTX)-resistant triple-negative breast cancer (TNBC) cells, TNFSF9 expression is elevated. Silencing TNFSF9 enhances PTX sensitivity, induces macrophage polarization from the M2 to the M1 phenotype, increases IL-1β and TNF-α secretion, and decreases IL-10 and TGF-β levels. Mechanistic analyses show that RBM15 upregulates TNFSF9 via m^6^A modification, while TNFSF9 overexpression rescues the suppressive effects of RBM15 silencing on PTX-resistant TNBC cells and xenograft tumors (Fu et al., 2025). Clinical specimens confirm RBM15-mediated TNFSF9 overexpression in PTX-resistant TNBC, suggesting that the RBM15/TNFSF9 axis is a key contributor to chemoresistance and immune modulation. In summary, RBM15 drives breast cancer progression by promoting metabolic reprogramming, stabilizing oncogenic transcripts, and conferring chemotherapy resistance, thereby representing a crucial therapeutic target in aggressive breast cancer subtypes.

### 4.5 Kidney cancer

In clear cell renal cell carcinoma (ccRCC), RBM15 is highly expressed and promotes cell proliferation, colony formation, migration, invasion, and EMT. This upregulation is largely attributed to histone H3 acetylation at the RBM15 promoter mediated by EP300/CBP. Mechanistic studies demonstrate that RBM15 stabilizes CXCL11 mRNA in an m^6^A-dependent manner, thereby enhancing macrophage infiltration and M2 polarization, which collectively promote tumor progression (Zeng et al., 2022). These findings reveal a critical EP300/CBP–RBM15–CXCL11 signaling axis in ccRCC and suggest a potential therapeutic strategy. Furthermore, natural compounds may modulate RBM15-mediated tumor regulation. Resveratrol has been shown to regulate ccRCC cell senescence by modulating RBM15-mediated CCNB1 mRNA stabilization while simultaneously suppressing EP300/CBP expression (Chen et al., 2023). *In vitro* and *in vivo* studies confirm the tumor-suppressive effects of resveratrol, suggesting its potential as a therapeutic agent targeting RBM15-dependent pathways in ccRCC. In summary, RBM15 promotes ccRCC progression through epigenetic activation and m^6^A-dependent stabilization of oncogenic transcripts, while resveratrol emerges as a promising RBM15-targeted therapeutic approach.

### 4.6 Cervical cancer

In cervical cancer (CC) tissues, RBM15 is highly expressed, and its elevated levels are associated with malignant tumor characteristics. Functional studies demonstrated that RBM15 knockdown suppresses CC cell proliferation, migration, and invasion, as well as tumor growth *in vivo*. Mechanistically, RBM15 promotes CC progression by installing m^6^A modification on the tumor suppressor DCN mRNA, thereby reducing DCN expression (Wang et al., 2025b); conversely, RBM15 silencing enhances DCN expression, while DCN depletion reverses this tumor-suppressive effect. In both CC and cervical squamous cell carcinoma/adenocarcinoma (CESC), OTUB2 is highly expressed, with levels progressively increasing during disease progression and correlating with poor prognosis. Functional assays revealed that OTUB2 knockdown suppresses CC cell proliferation and migration while promoting apoptosis. Mechanistically, RBM15-mediated m^6^A modification upregulates OTUB2 mRNA expression, which in turn activates the AKT/mTOR signaling pathway and drives malignant behaviors (Song and Wu, 2023). The AKT/mTOR agonist SC-79 partially reverses the inhibitory effect of OTUB2 silencing, highlighting the critical role of the RBM15/OTUB2/AKT/mTOR axis in CC progression. In CC tissues and cells, RBM15 is upregulated and preferentially enriches m^6^A modifications on the long non-coding RNA HEIH, enhancing its RNA stability (Quan et al., 2024). As an oncogenic lncRNA, HEIH sponges miR-802 to upregulate EGFR expression, thereby promoting CC cell proliferation, migration, and stemness, ultimately enhancing tumor growth. Overall, RBM15 drives CC progression by stabilizing HEIH expression and regulating CC cell proliferation, metastasis, and stem-like properties. RBM15 expression is also elevated in CC and associated with poor prognosis. Functional experiments showed that RBM15 knockdown inhibits CC cell proliferation and EMT. Mechanistically, RBM15 enhances m^6^A modification and translation of EZH2, which subsequently binds to the fibronectin 1 (FN1) promoter to form the RBM15/EZH2/FN1 signaling cascade, driving CC cell proliferation and EMT. EZH2 overexpression rescues the inhibitory effect of RBM15 silencing, suggesting that the RBM15/EZH2/FN1 axis plays a pivotal role in CC progression and may represent a potential therapeutic target (Wang and Tan, 2024). In HPV-related CC, expression of the m^6^A “writer” complex components METTL3, RBM15, and WTAP is upregulated, with RBM15 being particularly prominent. Studies revealed that HPV-E6 suppresses autophagy, thereby blocking RBM15 protein degradation and leading to its intracellular accumulation, while RBM15 mRNA levels remain unchanged (Nie et al., 2023). RBM15 binds to c-myc mRNA, increasing its m^6^A modification and protein expression, thereby promoting CC cell proliferation. RBM15 inhibition or blockade of m^6^A modification (e.g., cycloleucine treatment) reverses this proliferative effect. Collectively, HPV-E6 enhances RBM15 protein stability by suppressing autophagy, which in turn strengthens c-myc m^6^A modification and expression, driving CC cell growth.

### 4.7 Prostate cancer

In prostate cancer (PCa) with wild-type p53, the long non-coding RNA FTO-IT1 is upregulated, and its high expression is associated with poor survival. Mechanistically, FTO-IT1 directly binds RBM15 and inhibits RBM15-mediated m^6^A methylation and stabilization of p53 target gene mRNAs (such as FAS, TP53INP1, and SESN2), thereby blocking p53 signaling and suppressing cell cycle arrest and apoptosis. FTO-IT1 knockout restores m^6^A levels and expression of p53 target genes, suppressing PCa cell growth (Zhang J. et al., 2023). This indicates that FTO-IT1 attenuates p53 tumor-suppressive activity by repressing RBM15-mediated m^6^A modification, representing a potential therapeutic target. In castration-resistant prostate cancer (CRPC) and prostate cancer stem cells (PCSCs), the pseudogene AZGP1P2 is downregulated, and its high expression correlates with longer progression-free survival. Functional studies showed that AZGP1P2 upregulation suppresses PCSC stemness and enhances paclitaxel sensitivity, thereby inhibiting tumor growth and metastasis (Wang et al., 2023). Mechanistically, AZGP1P2 binds to ubiquitin-activating enzyme E1 (UBA1) and RBM15, forming a complex that promotes ubiquitin-mediated RBM15 degradation, thereby regulating m^6^A modification and degradation of TPM1 mRNA. *In vivo* experiments using xenografts and patient-derived organoids confirmed that the AZGP1P2/UBA1/RBM15/TPM1 axis enhances paclitaxel efficacy (Wang et al., 2023). In PCa patients, 34 m^6^A-related prognostic lncRNAs were identified and classified into two subtypes based on expression and survival patterns. Seven of these lncRNAs (including LINC02666 and AC022211.1) are regulated by RBM15, and a prognostic risk model was established with strong predictive power for overall survival. RBM15 expression is closely correlated with PCa progression, survival, and immune responses (Hu et al., 2024). Patients with high RBM15 expression exhibit increased sensitivity to AMG-232, while RBM15 knockdown reduces PCa cell viability and promotes apoptosis, underscoring RBM15’s critical role in PCa progression and immunoregulation.

### 4.8 Pancreatic cancer

As a critical regulator of m^6^A methylation, RBM15 is aberrantly upregulated in various cancers and correlates with poor prognosis. Pan-cancer analysis revealed mutations or copy number variations of RBM15 in 25 cancer types, with high expression significantly associated with overall survival (OS), disease-free interval (DFI), progression-free interval (PFI), and disease-specific survival (DSS). In pancreatic adenocarcinoma (PAAD), RBM15 overexpression is closely linked to patient survival and shows positive correlation with immune infiltration and immune checkpoint markers (Dong et al., 2023). Functional assays demonstrated that RBM15 knockdown markedly suppresses PAAD cell proliferation. Protein interaction and pathway analyses indicated that RBM15 participates in multiple biological processes, including cell cycle regulation, sister chromatid cohesion, serine/threonine phosphorylation, and T-cell receptor signaling, suggesting its role as a prognostic biomarker and potential immunotherapy target in PAAD. Experimental studies further confirmed that RBM15 promotes PAAD cell proliferation, migration, and metastasis, and its expression is strongly associated with T-cell infiltration. Both *in vitro* and *in vivo* studies demonstrated that RBM15 silencing significantly inhibits PAAD growth and metastasis, underscoring its oncogenic role. Moreover, Irradiation of 125I seed radiotherapy suppresses glycolysis and tumor growth by inhibiting RBM15/KLF5-mediated m^6^A modification, thereby reducing proliferation and invasion while promoting apoptosis (Song et al., 2025). RBM15 overexpression partially reverses this effect, suggesting that the RBM15/KLF5 axis is a critical mediator of Irradiation of 125I radiotherapy efficacy in PAAD.

### 4.9 Esophageal squamous cell carcinoma

RBM15, as an m^6^A RNA methyltransferase, is upregulated in ESCC and promotes tumor proliferation and migration. Mechanistically, RBM15 facilitates m^6^A modification of pri-miR-3605–5p, enhancing its maturation into miR-3605–5p, which subsequently represses KRT4 expression. Restoration of KRT4 activates the p53 signaling pathway, counteracting RBM15-driven proliferation and migration (Wang, 2024).

### 4.10 Head and neck squamous cell carcinoma

In laryngeal squamous cell carcinoma (LSCC) patients, global mRNA m^6^A methylation levels are significantly elevated, with RBM15 upregulation correlating with poor prognosis. Functional studies showed that RBM15 knockdown inhibits LSCC cell proliferation, migration, and invasion while inducing apoptosis, whereas RBM15 overexpression reverses these effects. Mechanistically, RBM15 regulates TMBIM6 mRNA via m^6^A modification and enhances its stability through IGF2BP3, thereby promoting LSCC progression (Wang et al., 2021). In cisplatin-resistant laryngeal cancer (LC), RBM15 is upregulated, and its silencing decreases the IC50, suppresses cell viability and proliferation, elevates Fe^2+, reactive oxygen species (ROS), and malondialdehyde (MDA) levels, and increases ACSL4 expression, thereby inducing ferroptosis. Mechanistically, RBM15 stabilizes KDM5B mRNA in an IGF2BP3-dependent manner, leading to FER1L4 downregulation and GPX4 upregulation. KDM5B further upregulates KCNQ1OT1 and suppresses ACSL4, thus inhibiting ferroptosis. Overexpression of KDM5B/KCNQ1OT1 or knockdown of FER1L4 rescues ferroptosis suppression and enhances cisplatin resistance. *In vivo* experiments confirmed that RBM15 knockdown significantly suppresses tumor growth (Liang et al., 2024). Collectively, RBM15 promotes KDM5B expression to inhibit ferroptosis and regulate the FER1L4/GPX4 and KCNQ1OT1/ACSL4 signaling axes, thereby enhancing cisplatin resistance in LC and representing a potential therapeutic target.

### 4.11 Ovarian cancer

In ovarian cancer (OC) and paclitaxel (PTX)-resistant cells, RBM15 is upregulated and correlates with poor prognosis. RBM15 overexpression enhances cell viability and colony formation, decreases PTX sensitivity, and suppresses apoptosis, whereas RBM15 knockdown inhibits cell proliferation and tumorigenesis, increases PTX sensitivity and apoptosis, and reduces sphere-forming ability in resistant cells (Yuan et al., 2023). Mechanistically, RBM15 maintains MDR1 mRNA stability via m^6^A modification, upregulating MDR1 expression and promoting chemoresistance. Moreover, the TGF-β signaling pathway suppresses RBM15 expression, revealing a TGF-β/RBM15/MDR1 regulatory axis (Yuan et al., 2023).

### 4.12 Gastricr cancer

In gastric cancer, RBM15 expression is significantly elevated and associated with poor prognosis. Functional assays demonstrated that RBM15 promotes GC cell proliferation and invasion. Mechanistically, RBM15 regulates downstream target ATP citrate lyase (ACLY) expression via m^6^A modification, which is recognized by IGF2BP2, thereby activating ACLY, driving lipid biosynthesis, and enhancing tumor malignancy (Cai et al., 2025).

### 4.13 Osteosarcoma

In osteosarcoma, RBM15 has been implicated in circRNA-mediated m^6^A regulation of tumor metabolism and progression. circ-CTNNB1 is highly expressed in osteosarcoma tissues and predominantly localized in the nucleus. Functional experiments demonstrated that circ-CTNNB1 overexpression markedly promotes osteosarcoma cell proliferation, invasion, and metastasis. Mechanistically, circ-CTNNB1 interacts with RBM15 to promote m^6^A modification of key glycolytic enzymes—including hexokinase 2 (HK2), glucose-6-phosphate isomerase (GPI), and phosphoglycerate kinase 1 (PGK1)—thereby enhancing glycolytic activity and driving malignant progression (Yang et al., 2023). This study highlights the critical role of the circ-CTNNB1/RBM15/m^6^A axis in metabolic reprogramming and osteosarcoma progression, offering a potential therapeutic target.

### 4.14 Acute myeloid leukemia

RBM15 is an RNA-binding protein that plays a pivotal role in the initiation and maintenance of acute myeloid leukemia (AML). Functionally, RBM15 recruits the m^6^A methyltransferase complex (MTC) to specific RNA substrates, thereby regulating m^6^A deposition and influencing RNA stability, splicing, and translation. Through this mechanism, RBM15 modulates the fate of transcripts encoding key transcription factors and signaling molecules, ultimately impacting the self-renewal and differentiation balance of leukemia stem and progenitor cells. This study focuses on the recurrent t (1; 22) translocation in acute megakaryoblastic leukemia (AMKL), which encodes the RBM15-MKL1 (RM) fusion protein. The researchers proposed that RM may drive leukemogenesis by altering m^6^A modification (Mayday et al., 2025). RM not only retains the RNA-binding and m^6^A-modifying functions of RBM15 but also selectively regulates specific mRNA targets, particularly Frizzled genes in the Wnt signaling pathway. In a murine RM-AMKL model, treatment with the METTL3 inhibitor STM3675 (which reduces m^6^A deposition) induced apoptosis and significantly prolonged the survival of transplanted mice (Mayday et al., 2025). Further experiments demonstrated that RM upregulates Frizzled genes, while METTL3 inhibition downregulates them, indicating that this regulation is m^6^A-dependent. Direct knockdown of Frizzled genes also suppressed RM-AMKL growth both *in vitro* and *in vivo*, confirming that Wnt signaling is a key oncogenic driver. This study also reveals the mechanism of m^6^A deposition on chromatin-associated RNAs (caRNAs). It shows that the RNA-binding protein RBFOX2 functions as a chromatin factor that preferentially recognizes m^6^A-modified caRNAs and further recruits the m^6^A MTC component RBM15, thereby promoting promoter-associated RNA methylation (Dou et al., 2023a). Subsequently, RBM15 not only interacts with the m^6^A reader YTHDC1 but also recruits the Polycomb Repressive Complex 2 (PRC2) to RBFOX2-bound loci, resulting in locus-specific chromatin silencing and transcriptional repression (Dou et al., 2023a). In AML, this RBFOX2/m^6^A/RBM15/YTHDC1/PRC2 axis is essential for leukemia cell survival, proliferation, and the self-renewal and maintenance of leukemia stem/progenitor cells. Downregulation of RBFOX2 significantly suppresses AML cell growth and promotes myeloid differentiation.

## 5 Roles of RBM15 in tumor immunity

### 5.1 RBM15 regulates immune cell functions

**FIGURE 4:**
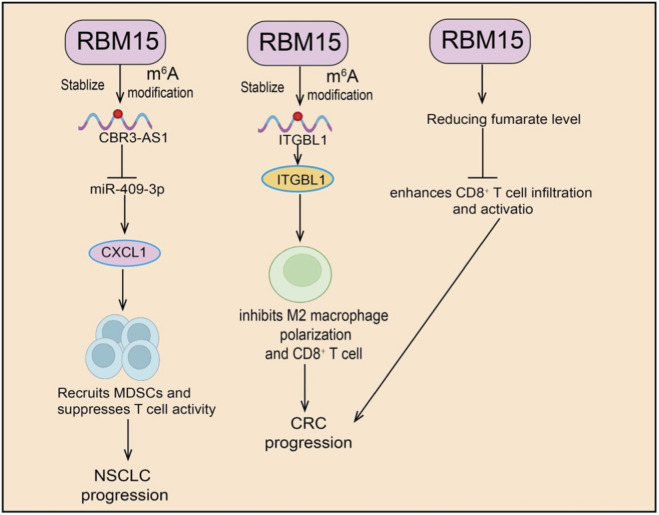
Functional roles of RBM15 in tumor immunity and progression. RBM15 stabilizes and promotes m^6^A modification of target RNAs. (Left) RBM15 stabilizes CBR3-AS1, leading to suppression of miR-409–3p and upregulation of CXCL1, which recruits MDSCs and suppresses T cell activity, thereby promoting NSCLC progression. (Middle) RBM15 stabilizes ITGBL1, suppressing M2 macrophage polarization and CD8^+^ T cell responses, contributing to CRC progression. (Right) RBM15 reduces fumarate levels, enhancing CD8^+^ T cell infiltration and activation, which modulates immune responses in CRC.

RBM15 plays a pivotal role in tumor immune regulation by modulating immune cell functions and remodeling the TME through multiple mechanisms. Evidence shows that RBM15 not only regulates the m^6^A modification of immune-related genes but also indirectly shapes antitumor immunity by affecting immune cell recruitment, polarization, and activity. In pancreatic cancer, RBM15 regulates immune cell infiltration in the TME via m^6^A modification, particularly influencing macrophage function. Knockdown of RBM15 suppresses pancreatic cancer cell growth while promoting macrophage infiltration and enhancing their phagocytic capacity against tumor cells, suggesting a pro-oncogenic role of RBM15 in pancreatic cancer progression and immune regulation, as well as its potential as a target for optimizing immunotherapy. In ESCC, procollagen-lysine two-oxoglutarate 5-dioxygenase 3 (PLOD3) is highly expressed and associated with favorable prognosis (Lin et al., 2024). Increased CD4^+^ T cell infiltration has been observed in tumors with high PLOD3 expression. Analyses revealed a strong correlation between RBM15 and PLOD3, with RBM15 loss reducing PLOD3 expression in ESCC cells. Together, RBM15 and PLOD3 form a prognostic gene signature for ESCC (Lin et al., 2024). Mechanistically, RBM15 promotes CD4^+^ T cell infiltration by regulating PLOD3, suggesting that both molecules may serve as independent prognostic factors in ESCC. In non-small cell lung cancer (NSCLC), RBM15 expression correlates positively with CBR3-AS1 expression and overall survival in patients receiving radiotherapy. Clinically, patients with high RBM15 expression are more prone to radioresistance and poor prognosis after radiotherapy. *In vitro*, silencing RBM15 enhances the inhibitory effects of radiotherapy on NSCLC proliferation and invasion while promoting apoptosis, whereas RBM15 overexpression has the opposite effect. Mechanistic studies revealed that RBM15 upregulates CBR3-AS1 through an m^6^A–IGF2BP3–dependent pathway. CBR3-AS1 sponges miR-409–3p to elevate CXCL1 expression, which in turn recruits MDSCs and suppresses T cell activity (Hu et al., 2025). *In vivo*, silencing RBM15 decreases MDSC infiltration, enhances CD4^+^ and CD8^+^ T cell infiltration, and reverses radioresistance (Figure 4). Bioinformatics analyses further identified a prognostic model based on RBM15 and YTHDC1, which effectively stratifies high- and low-risk patients. Immune profiling suggested that RBM15 negatively correlates with γδ T cells and positively correlates with activated natural killer (NK) cells, while drug sensitivity analysis revealed diverse associations with multiple therapeutic agents. In COAD, integrin beta-like 1 (ITGBL1) and RBM15 are both upregulated. Silencing ITGBL1 inhibits proliferation, migration, invasion, M2 macrophage polarization, and CD8^+^ T cell immunosuppression, while RBM15 deficiency significantly reduces tumor growth (Zhu et al., 2025) (Figure 4). Mechanistic studies showed that RBM15 promotes COAD progression and immunosuppression by upregulating ITGBL1 expression through m^6^A modification. In breast cancer, both RBM15 and karyopherin subunit alpha 2 (KPNA2) are significantly overexpressed in tissues and cell lines. Knockdown of RBM15 suppresses proliferation, migration, and invasion of breast cancer cells *in vitro*, while enhancing lymphocyte immune activity. *In vivo*, RBM15 loss markedly inhibits tumor growth. Mechanistic studies demonstrated that RBM15 stabilizes KPNA2 mRNA via m^6^A modification, thereby promoting breast cancer progression (Wang et al., 2025a). Collectively, RBM15 precisely regulates immune-related genes and non-coding RNAs through m^6^A modification, modulating the functions of MDSCs, macrophages, and T cells. By reshaping the TME, RBM15 facilitates immune evasion and drives tumor progression.

### 5.2 Potential links between RBM15 and immunotherapy

Immune checkpoint blockade (ICB) therapy has shown promise in treating advanced CRC, particularly in patients with high microsatellite instability (MSI-H). However, only a subset of patients respond effectively, highlighting the urgent need to enhance immunotherapy efficacy. Studies revealed that RBM15 is highly expressed in CRC and correlates with poor prognosis. Functional experiments demonstrated that RBM15 deficiency increases fumarate hydratase (FH) expression, thereby reducing fumarate levels. As fumarate is a known inhibitor of antitumor immune responses (Wang C. et al., 2025) (Figure 4), this metabolic reprogramming alleviates immunosuppression. *In vivo*, RBM15 loss significantly delays tumor progression and enhances CD8^+^ T cell infiltration and activation within the TME. Taken together, these findings suggest that RBM15 regulates FH/fumarate metabolism to shape the immune microenvironment in CRC and may represent a promising therapeutic target to improve ICB efficacy (Wang C. et al., 2025).

## 6 Advantages and challenges of targeting RBM15 in cancer therapy

Targeting RBM15 holds significant therapeutic advantages in oncology. First, as an m^6^A “writer” enzyme, RBM15 exhibits substrate specificity, enabling selective modification of particular RNAs. This substrate preference allows targeted modulation of oncogenic gene expression while minimizing off-target effects on normal RNAs, thereby reducing potential side effects (Liang et al., 2023). Second, RBM15 is aberrantly upregulated in various cancers but displays relatively low expression in normal tissues. This expression disparity provides a rationale for selective therapeutic targeting, conferring tumor specificity and distinguishing pathological states from physiological ones.

However, to date, no small-molecule inhibitors directly targeting RBM15 have entered clinical development, primarily due to its functional complexity and the limited research in this area. On one hand, RBM15 is broadly involved in normal cellular physiology, and its direct inhibition may pose a risk of severe side effects such as bone marrow suppression (Hu et al., 2016). In contrast, targeting downstream effectors of RBM15, such as IGF2BP3, which are closely associated with specific oncogenic pathways, could reduce systemic toxicity (Hu et al., 2025). Consequently, the development of RBM15 inhibitors still lacks sufficient preclinical and clinical evidence, which in turn limits its feasibility as a prioritized drug target.

Nevertheless, challenges remain. Although the role of RBM15 in cancer initiation and progression has been well established, its mechanisms in immune regulation are still poorly understood. Current evidence suggests that RBM15 may modulate the TME by influencing immune cell infiltration or regulating immunosuppressive factors, but the precise pathways and interaction patterns remain unclear (Wang C. et al., 2025). This knowledge gap limits the development of RBM15-based immunotherapeutic strategies and underscores the need for further mechanistic studies in tumor immunology. RBM15 exhibits advantages as a therapeutic target due to its RNA substrate specificity and tumor-selective expression. However, incomplete understanding of its role in immune regulation presents a major challenge, highlighting the necessity for deeper mechanistic research to advance clinical translation.

## 7 Clinical applications and translational prospects

With the rapid progress of epitranscriptomics, RBM15 has emerged as a clinically relevant RNA-binding protein and m^6^A regulator. At the biomarker level, accumulating evidence indicates that aberrant RBM15 expression strongly correlates with patient prognosis across multiple cancers. For instance, RBM15 upregulation in acute myeloid leukemia, hepatocellular carcinoma, and breast cancer is associated with accelerated tumor progression and unfavorable outcomes, whereas its downregulation in certain solid tumors may reflect impaired tumor-suppressive pathways (Bhat et al., 2024). Thus, RBM15 holds promise as a diagnostic and prognostic biomarker to aid risk stratification and guide personalized treatment decisions. In therapeutic development, targeting RBM15 and its m^6^A-mediated modifications represents a novel strategy. Modulating RBM15 activity with small-molecule inhibitors or RNA interference could restore the balance between oncogenes and tumor suppressors, thereby inhibiting tumor growth and metastasis. Given RBM15 involvement in immune pathways, its inhibition may also mitigate immune evasion and enhance the efficacy of immunotherapies such as immune checkpoint inhibitors. However, no specific drugs directly targeting RBM15 have yet been developed, and the feasibility of drug design requires further exploration.

Despite its potential, several challenges remain. First, RBM15 may act as either an oncogene or tumor suppressor depending on cancer type and context, necessitating detailed characterization of its context-dependent functions. Second, RBM15 may undergo phase separation to form nuclear or cytoplasmic condensates, regulating RNA processing in a spatiotemporal manner, which remains incompletely understood. Third, questions regarding its substrate specificity and direct RNA targets are unresolved, as are its interactions with other RBPs and m^6^A regulators. Additionally, whether RBM15 modulates chromatin-associated RNAs to influence 3D genome organization is an intriguing open question. Importantly, its role in immune regulation and TME remodeling requires clarification to inform combination therapies with immunotherapeutic agents.

This review has several limitations. First, studies investigating the role of RBM15 in tumor immunity are still limited, which makes the discussion on its immunoregulatory mechanisms relatively insufficient. Second, no specific inhibitors or small molecules targeting RBM15 have been developed so far, restricting in-depth discussion on its potential application in targeted therapy or combination with PD-L1 immunotherapy.

## 8 Conclusion and future perspectives

As an RNA-binding protein and m^6^A regulator, RBM15 exerts multifaceted functions in cancer progression. Through both m^6^A-dependent and -independent mechanisms, RBM15 modulates RNA stability, splicing, transport, and translation, thereby regulating the balance between oncogenes and tumor suppressors. In addition, RBM15 cooperates with epigenetic factors and transcriptional complexes to control key processes such as proliferation, invasion, and metabolic reprogramming. In tumor immunology, RBM15 shapes immune responses by regulating immune-related molecules and signaling pathways, influencing the TME, and either promoting or suppressing immune evasion (Wang C. et al., 2025). Thus, RBM15 not only drives intrinsic malignant behavior of cancer cells but also acts as a critical regulator of antitumor immunity.

Future research should clarify the tissue- and context-specific functions of RBM15, define its substrate selectivity, and explore its phase separation dynamics and potential roles in chromatin-associated RNA regulation and 3D genome organization. Clinically, RBM15 shows promise as both a diagnostic/prognostic biomarker and a therapeutic target. Targeting RBM15 or its m^6^A modifications may directly suppress malignant phenotypes while synergizing with immunotherapies such as checkpoint inhibitors, thereby opening new avenues for precision oncology. RBM15 represents a central link between epitranscriptomic regulation and cancer biology. Continued research integrating molecular mechanisms, clinical data, and immunological insights will be essential for harnessing RBM15 as a biomarker and therapeutic target, ultimately advancing precision cancer therapy.
